# Activity of Tea Tree (*Melaleuca alternifolia*) Essential Oil against L3 Larvae of *Anisakis simplex*

**DOI:** 10.1155/2014/549510

**Published:** 2014-05-25

**Authors:** Carlota Gómez-Rincón, Elisa Langa, Paula Murillo, Marta Sofía Valero, César Berzosa, Víctor López

**Affiliations:** Facultad de Ciencias de la Salud, Universidad San Jorge, Campus Universitario Villanueva de Gállego, Autovia A-23 Zaragoza, Huesca, Km 299, 50.830 Villanueva de Gállego, Zaragoza, Spain

## Abstract

Nematicidal activity of *Melaleuca alternifolia* essential oil, commonly known as tea tree oil (TTO), was assayed *in vitro* against L3 larvae of *Anisakis simplex*. The results showed a mortality of 100% for concentrations between 7 and 10 **μ**L/mL after 48 h of incubation, obtaining an LD50 value of 4.53 **μ**L/mL after 24 hours and 4.27 **μ**L/mL after 48 hours. Concentration-dependent inhibition of acetylcholinesterase was observed for tea tree essential oil showing inhibition values of 100% at 100 **μ**L/mL. This fact suggests that TTO may act as an AChE inhibitor. Terpinen-4-ol was discarded as main larvicide compound as it did not show larvicidal or anticholinesterase activity. The data obtained suggest that the essential oil of *Melaleuca alternifolia* may have a great therapeutic potential for the treatment of human anisakiasis.

## 1. Introduction 

In the last years, the implication of* Anisakis simplex* in gastric, intestinal, and allergic clinical disorders has significantly increased [1]. More than 90% of anisakiasis cases are reported in Japan [2]. However, the high human consumption together with the high* Anisakis* prevalence in seafood from coastal areas of Europa [3, 4] suggests that anisakiasis may be an underdiagnosed illness [1–3, 5–7].

Larval endoscopic extraction is the preferred treatment of gastric and intestinal anisakiasis but if the larva is not accessible surgical treatment should be necessary [1]. Pharmacological treatments with antibiotics, anticholinergics, and/or corticosteroids have been occasionally used but their efficacy remains unclear [6, 8, 9]. On the other hand, some studies have suggested that treatment with nematicides as albendazole may constitute an effective therapy [10–13]. However, the worldwide development of resistance to chemical anthelmintic drugs [14] and a current social demand for natural therapeutic products has increased the search for new biocide molecules. Plant products are the basis of traditional medicine and the only health resource available in many communities from developing countries. Otherwise, several plant essential oils and their derived compound have shown biocidal activity against* Anisakis *spp. [15–19].

Tea tree oil (TTO) is the essential oil obtained by hydrodistillation from* Melaleuca alternifolia*, a species native from New South Wales, Australia [20]. Tea tree oil is a natural product derived from the Australian native plant* Melaleuca alternifolia* that has shown multiple biological activities such as anti-inflammatory, antitumoral, and biocidal properties [21]. Tea tree oil is an effective bactericide [22], fungicide [23], antiviral [24], and insecticidal agent [25, 26]. Furthermore, several studies have demonstrated its effectiveness against protozoa parasites as* Leishmania major* or* Trypanosoma brucei* [27]. But there is no evidence of the effect that this substance might have on parasite nematodes.

Many nematicides act through an inhibition of parasite motility. The motility blockade causes inability to remain on the host favouring the parasite elimination. One of the mechanisms affecting this parasite function is the effect on acetylcholine and/or acetylcholinesterase activity. Levamisole acts as an agonist on nicotinic acetylcholine receptors at the nematode neuromuscular junction, leading to sustained neuromuscular depolarization and spastic paralysis [28, 29]. In this line, Mills et al. [30] propose that the pediculicide effect of TTO could be due to, at least in part, anticholinesterase activity of its two main components terpinen-4-ol and 1,8 cineole.

The aim of the present study was to analyze the activity of tea tree essential oil and its main component terpinen-4-ol against* Anisakis simplex* third stage larvae (L3) and to explore the inhibition of acetylcholinesterase as possible mechanism of action.

## 2. Materials and Methods 

### 2.1. Essential Oil

Tea tree essential oil was supplied by Pranarôm. Although the essential oils are chemically characterized by Pranarôm International, they were analysed by GC-MS on an Agilent 6890N Network GC system coupled to a 5973 Network Mass Selective Detector, accelerating voltage −69.9 eV, recoding masses of 35.00–400.00. GC conditions are as follows: injector temperature: 150°C; temperature programme: starts from 50°C, 20°C/min to 300°C; column: HP5MS (5% phenylmethylsiloxane) capillary, 30.0 m × 250 *μ*L × 0.25 *μ*m nominal; carrier gas: helium at 1.0 mL/min. A NIST library was used for comparison of MS data.

### 2.2. *In Vitro* Larvicidal Activity

*Anisakis simplex* L3 was isolated from the intermediary host* Micromesistius poutassou* (blue whiting) purchased from several fish markets in Zaragoza. Worms were washed several times with sterile solution of 0.9% NaCl and identified under light microscope according to morphological features [31, 32]. Only intact* Anisakis simplex s.l*, L3 with length > 2.0 cm were used. Ten larvae were introduced in each well of polystyrene plates with 2 mL of sterile saline solution containing different concentration of the test solutions. The final concentrations of* Melaleuca alternifolia* essential oil were: 10, 7, 5, 4, 3, 2, 1, and 0.5 *μ*g/mL. For terpinel-4-ol the final concentrations tested were 0.1, 1, and 10 *μ*g/mL. The parasites were incubated at 37°C in 5% CO_2_. Each dilution, together with saline solution negative control, was tested by triplicates on three different days. Levamisole (100 *μ*L/mL) was used as the reference antiparasitic drug. Larvae were examined at 24 h and 48 h under microscope and immobile L3 were considered dead. In order to evaluate the biocidal activity of the samples, an average mortality was determined. LC50 was calculated using nonlinear regression (GraphPad Prism 5).

### 2.3. Inhibition of Acetylcholinesterase

The inhibition of acetylcholinesterase (AChE) was determined by the Ellman method [33] with some modifications. The AChE activity was measured using a 96-microplate, each well containing 25 *μ*L of 15 mM ATCI in Millipore water, 125 *μ*L of 3 mM DTNB in buffer C (50 mM Tris-HCl, pH 8, 0.1 M NaCl, 0.02 M MgCl_2_6H_2_O), 50 *μ*L buffer B (50 mM Tris-HCl, pH 8, 0.1% bovine serum), and 25 *μ*L of test compound. Every concentration tested of TTO (0.1, 1, 10, and 100 *μ*L/mL), terpinen-4-ol (0.1; 1 and 10 *μ*L/mL), and Levamisole (0.1, 1, 10, and 100 *μ*L/mL) was diluted in DMSO and tested in triplicates. Then, 25 *μ*L 0.22 U/mL AChE was added and the absorbance was measured eight times every 13 s at 405 nm.

### 2.4. Statistical Analyses

Data were subjected to analysis of variance, and mean comparison was performed by one-way ANOVA plus Scheffé multiple comparisons (*P* ≤ 0.05). The statistical analysis was performed using PASW Statistics 18 program.

## 3. Results 

### 3.1. Composition of* Melaleuca alternifolia* Essential Oil

Eight main compounds were detected in the essential oil analyzed by GC-MS (Table 1). The compounds and their abundance are consistent with the chemical characterization provided by Pranarôm International.

### 3.2. *In Vitro* Larvicidal Activity

Tea tree essential oil showed a significant dose-dependent lethal effect on* Anisakis simplex* L3 (*P* < 0.05) (Table 2). Concentrations of 5 *μ*L/mL and above impair the larvae survival after 24 h exposure (*P* ≤ 0.05). Otherwise, 4 *μ*L/mL of TTO required 48 h to reduce larval vitality. The most effective concentrations were 10 *μ*L/mL, which showed a total lethal effect at 24 h and 7 *μ*L/mL that caused 93% and 100% mortality after 24 h and 48 h incubation. Terpinen-4-ol did not show any larvicide effect at tested concentration. Levamisole, an anthelmintic drug used as positive control, was highly effective causing a 100% larval mortality after 24 h.

**Figure 1:**
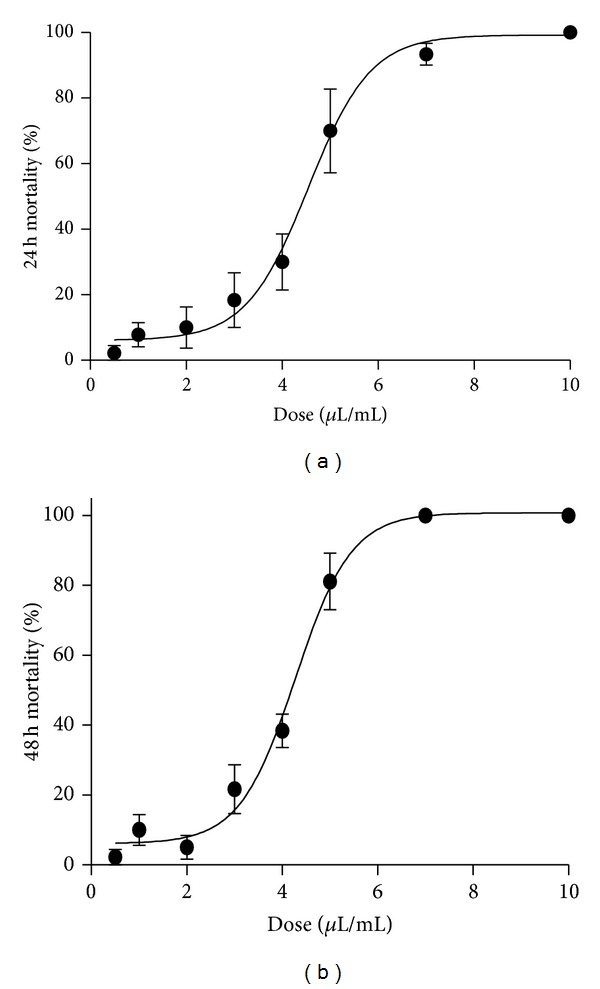
Dose-response effect of* Melaleuca alternifolia *essential oil after 24 and 48 h of exposure.

The logistic regression of data revealed a dose-response effect for the TTO that showed LD_50_ values of 4.53 and 4.27 *μ*L/mL after 24 and 48 h (Figure 1).

### 3.3. Inhibition of Acetylcholinesterase Assay

Concentration-dependent inhibition of AChE was observed for tea tree essential oil and levamisole (Table 3). TTO showed inhibition values of 100% at 100 *μ*L/mL and were significantly more effective than the AChE inhibitor levamisole at the same concentration (*P* ≤ 0.05). However, terpinen-4-ol, the main component of TTO, did not show inhibition of AChE at the tested concentrations.

## 4. Discussion 

The increasing worldwide incidence of anisakiasis together with the lack of effective pharmacological treatments warrants the search for new active molecules. Although in most cases anisakiosis resolved spontaneously, the severity of potential complications such as peritonitis or intestinal wall perforations, frequently surgical treatments are required [34]. On the other hand, in the current context of economic crisis, optimization of health resources is essential. Therefore the search for less invasive and expensive sanitary interventions should be a research priority in health sciences. In that sense, the use of essential oils and natural products could provide a noninvasive, inexpensive, and effective treatment for human anisakiasis.

In recent years, the biocidal activity against *Anisakis* L3 of several essential oils and some of its components has been studied. Romero et al. [19] demonstrated that 125 *μ*g/mL of* Matricaria chamomilla* essential oil induced 100% larval mortality after 4 h* in vitro* and reduced the pathogenic effects in experimentally infected rats. The results obtained in our study show that TTO was effective, obtaining significant larvicidal effect at doses over 4 *μ*L/mL. The maximal concentration tested (10 *μ*L/mL) was 100% lethal at 24 h. Similarly, at a concentration of 7 *μ*L/mL, larvicidal activity was 93% at 24 h and 100% after 48 h of essential oil exposure. Observed mortality for other concentrations was low; however, mortality percentages were 52% and 83% after 24 and 48 h of exposure to 5 *μ*L/mL of EO.

Many studies have demonstrated the potent biocide effect of TTO. Many bacteria are susceptible to concentrations of 1% or less even against antibiotic-resistant strains [21]. TTO has also a fungicidal activity at concentrations ranging from 0.12 to 2% against yeast and dermatophytes [23, 35]. Although there are no previous references regarding the TTO LD50 against nematodes, the values obtained in our study (LD50_24 h_ = 4.53 *μ*L/mL and LD50_48 h_ = 4.14 *μ*L/mL) suggest that TTO could be an effective nematicide.

Essential oils are complex mixtures of compounds from natural origin, among which the terpenes have been largely studied and have shown useful pharmaceutical properties. Several monoterpenes as carvacrol, geraniol, or citronellol (monoterpenes) [19] and sesquiterpenes as nerolidol or farnesol [36] show a high potential against* Anisakis* L3. The main component of the essential oil obtained from tea tree is terpinen-4-ol [37]. Different authors suggest that this monoterpene is responsible, at least in part, for the antiprotozoal [27] and bactericidal [38] effects of the tea tree. Furthermore, recent studies suggest that terpinen-4-ol inhibits the motility of the plant parasitic nematode* Meloidogyne incognita* [39]. However, the larvicidal effect observed in our study cannot be associated with this compound since we did not observe any effect on* Anisakis* larvae. Therefore, we can deduce that the effect could be related to other compounds. It seems unlikely that alpha-terpinene and 1,8 cineole are responsible for the antiparasitic activity, at least individually, since they are inactive at high concentration against the plant nematode* Meloidogyne incognita* [39]. Otherwise, a possible candidate may be alpha-pinene (3% concentration in our essential oil), whose activity has been recently demonstrated* in vitro* and* in vivo* against* Anisakis *spp. [17]. However, due to the chemical complexity of essential oils, it should be noted that many of their properties are due to the synergistic or complementary effects of several components.

In this work we have also evaluated the AChE inhibition as a possible mechanism of action involved in the TTO nematicidal effect. Data showed that, at the same concentrations, TTO was a more effective AChE inhibitor than levamisole. These differences could be due to the fact that both drugs block the enzyme activity at different levels. Levamisole is a potent cholinergic agonist that binds preferentially to L-subtype nicotinic acetylcholine receptors in body-wall muscle causing hypercontracted paralysis, usually followed by relaxation and death [28, 29, 40, 41]. However, our results suggest that levamisole also has a moderate direct inhibitory effect on the enzyme. In this sense, tea tree essential oil seems to have a direct effect on the enzyme activity which is completely blocked at concentrations of 100 *μ*L/mL. These results suggest that inhibition of AChE could be a possible TTO mechanism of action against* Anisakis*. This hypothesis is consistent with the TTO anticholinesterase activity observed by Mills et al. [30] who proposed the competitive inhibition of AChE by 1,8 cineole and terpinen-4-ol to explain the TTO insecticidal effect. However, the IC50 values were 10.30 mM and 0.04 mM for terpinen-4-ol and 1,8 cineole, respectively, concluding that both compounds are weak inhibitors of the activity of the enzyme. In our study, even though the used concentration values were similar to those used by Mills et al. [30], terpinen-4-ol did not show anticholinesterase activity. This fact, together with the absence of larvicidal effect* in vitro*, leads us to discard terpinen-4-ol as main active compound against* Anisakis* L3. However, several studies suggest that the antimicrobial activity of TTO is attributed mainly to terpinen-4-ol [38]. For this reason, we are unable to discard that this compound has an active role in anthelmintic activity, as it may contribute indirectly or synergistically to the activity of other components of TTO.

Due to its chemical complexity, it is difficult to establish the mechanism of action involved in the biological effects of the essential oils. Therefore, the anticholinesterase effect observed in our study might be only partially responsible for the observed anthelmintic activity. It has been demonstrated that the interaction between the components of the TTO and cell membranes induces biochemical changes causing structural and functional cell integrity loss [21]. To our knowledge, there are no data about the effect of TTO on* Anisakis simplex* although recent studies suggest that essential oils or their constituents could cause alterations in the cuticle, muscle cells, and digestive system of L3. These effects would unleash the parasite death [16, 42] and, moreover, reduce the larval infectivity and their pathogenic effect* in vivo* [19].

Tea tree essential oil has long been used topically as an antiseptic; however, there are few data about its oral safety. Published data indicated that TTO can be toxic if ingested in higher doses [43].* In vivo* rat models toxicity studies showed LD50 values of 1.9–2.6 mL/kg [44]. Recent studies confirmed these data and revealed cytotoxic effects on human oral epithelial cells at concentrations of 500 *μ*L/mL [45]. Our* in vitro* experiments showed a high effectiveness of TTO against* Anisakis* L3 at lower concentrations suggesting that this molecule could be an effective nematicide. However, these results must be supported by* in vivo* studies to ensure the efficacy and safety of essential oil of tea tree in treating clinical anisakiasis.

## 5. Conclusions 

According to our results* Melaleuca alternifolia* essential oil showed a remarkable* in vitro* nematicidal effect for concentrations between 7 and 10 *μ*L/mL. Concentration-dependent inhibition of acetylcholinesterase was observed, suggesting that TTO may act as an AChE inhibitor. Terpinen-4-ol was discarded as main larvicide compound. Data obtained suggest that the essential oil of* Melaleuca alternifolia* may be a therapeutic tool for human anisakiasis.

## Figures and Tables

**Table 1:** Composition of *Melaleuca alternifolia* essential oil (% v/w).

Compound	%
*alpha*-Pinene	3.0
*alpha*-Terpinene	10.3
*Para*-cymene	5.9
Eucalyptol (1,8 cineole)	3.6
*gamma*-terpinene	22.5
Terpinolene	3.8
Terpinen-4-ol	46.9
*alpha*-Terpineol	3.8

**Table 2:** *In vitro* effect of *Melaleuca alternifolia* essential oil and terpinen-4-ol on L3 of *Anisakis simplex* survival. Results are expressed as average mortality ± standard deviation.

Compound	Concentration (*μ*L/mL)	Average mortality (%) (*n* = 90 L3)	
		24 h	48 h
Control		3.3 ± 10	5.6 ± 6.7

*Melaleuca alternifolia* EO	10	100*	100*
	7	93 ± 5.8*	100*
	5	52.3 ± 38.4*	81.1 ± 24.2*
	4	30 ± 20.9	38.3 ± 11.7*
	3	18.3 ± 20.4	21.7 ± 17.2
	2	10 ± 15.5	5 ± 8.4
	1	7.7 ± 10.9	10 ± 13.2
	0.5	2.2 ± 6.8	2.2 ± 6.8

Terpinen-4-ol	10	0	0
	1	0	0
	0.1	0	0

*Indicates values that differ from the control (*P* < 0.05).

Levamisole was used as positive control reference drug at the dose of 100 *μ*g/mL obtaining 100% of mortality after 24 h.

*n*: total number of L3 used to test every concentration.

**Table 3:** Percentage of acetylcholinesterase inhibition by *Melaleuca alternifolia* essential oil, terpinen-4-ol, and levamisole.

Compound	Concentration (*μ*L/mL)	% Inhibition
*Melaleuca alternifolia *essential oil	0.1	19.8 ± 2.9
	1	31.9 ± 26.5
	10	96.5 ± 18.9
	100	100

Terpinen-4-ol	0.1	0
	1	0
	10	0

Levamisole	1	0
	10	24.0 ± 5.9
	100	85.2 ± 2.1
